# What parents think about being approached about children’s trials, how this differs from what practitioners expect, and what this tells us about enhancing recruitment

**DOI:** 10.1186/1745-6215-12-S1-A116

**Published:** 2011-12-13

**Authors:** Bridget Young, Valerie Shilling, Helen Hickey, Emma Sowden, Rosalind L Smyth, Paula R Williamson

**Affiliations:** 1 Mental and Behavioural Health Science, University of Liverpool, Liverpool, L69 3GB, UK; 2 Child Health Research Group, Peninsula Medical School, University of Exeter, PL6 8BU, UK; 3 Biostatistics, University of Liverpool, Liverpool, L69 3GS, UK; 4 Reproductive and Developmental Medicine, University of Liverpool, Liverpool, L12 2AP, UK

## Objectives

Recruiting to clinical trials that involve vulnerable patient groups such as children is regarded as challenging. We compared how parents and practitioners described their experiences of recruitment to clinical trials of medicines for children in order to identify strategies to optimise recruitment and its conduct.

## Methods

Qualitative study of recruitment within four randomised controlled trials at 11 research sites in the UK. We compared i) audio-recording of practitioner-family dialogue during trial recruitment discussions with ii) audio-recordings of interviews that we subsequently conducted with parents and practitioners. Analyses of transcribed audio-recordings were informed by the principles of the constant comparative method.

## Results

Parents from 59 families were interviewed, 41 of whom had participated in audio-recorded recruitment discussions. Thirty one practitioners (doctors and nurses) were interviewed. While parents said little in the recruitment discussions and asked few questions, they reported a positive experience of the trial approach describing a sense of comfort and safety. Parents wanted to be told about a trial if their child was eligible - they did not want practitioners to exclude their child without discussing it. Even if parents declined or if the discussion took place at a difficult time, they understood the need to approach them and spoke of the value of research. Some parents viewed research participation as an ‘exciting’ opportunity. By contrast, practitioners often worried about approaching families about research in case it burdened them. Some practitioners were particularly apprehensive about approaching families and implied that recruiting to clinical trials was something which they found aversive. Many were also concerned about the amount of information they had to provide and believed this overwhelmed families.

## Conclusions

The concerns of some practitioners, that parents would be overburdened, stood in sharp contrast to the perspectives of parents. Contrary to what practitioners often expected, parents were positive about being approached to enter their child into a clinical trial. Helping practitioners to understand how families perceive clinical trials and providing them with ‘moral’ support in approaching families may enhance recruitment to children’s clinical trials. This strategy may also potentially benefit recruitment in trials with other vulnerable patient groups [1].
